# Weakly Supervised Polyp Segmentation in Colonoscopy Images Using Deep Neural Networks

**DOI:** 10.3390/jimaging8050121

**Published:** 2022-04-22

**Authors:** Siwei Chen, Gregor Urban, Pierre Baldi

**Affiliations:** 1 Department of Computer Science, University of California, Irvine, CA 92697, USA; siweic@uci.edu (S.C.); gurban@uci.edu (G.U.); 2 Institute for Genomics and Bioinformatics, University of California, Irvine, CA 92697, USA; 3 Center for Machine Learning and Intelligent Systems, University of California, Irvine, CA 92697, USA

**Keywords:** machine learning, deep learning, convolutional neural networks, colorectal cancer, colonoscopy quality improvement

## Abstract

Colorectal cancer (CRC) is a leading cause of mortality worldwide, and preventive screening modalities such as colonoscopy have been shown to noticeably decrease CRC incidence and mortality. Improving colonoscopy quality remains a challenging task due to limiting factors including the training levels of colonoscopists and the variability in polyp sizes, morphologies, and locations. Deep learning methods have led to state-of-the-art systems for the identification of polyps in colonoscopy videos. In this study, we show that deep learning can also be applied to the segmentation of polyps in real time, and the underlying models can be trained using mostly weakly labeled data, in the form of bounding box annotations that do not contain precise contour information. A novel dataset, Polyp-Box-Seg of 4070 colonoscopy images with polyps from over 2000 patients, is collected, and a subset of 1300 images is manually annotated with segmentation masks. A series of models is trained to evaluate various strategies that utilize bounding box annotations for segmentation tasks. A model trained on the 1300 polyp images with segmentation masks achieves a dice coefficient of 81.52%, which improves significantly to 85.53% when using a weakly supervised strategy leveraging bounding box images. The Polyp-Box-Seg dataset, together with a real-time video demonstration of the segmentation system, are publicly available.

## 1. Introduction

Colorectal cancer (CRC) is the third most lethal and third most commonly diagnosed cancer in the United States [1]. Most CRC cases arise from growths of adenomatous polyps, most of which are benign but can become malignant over time, leading to death unless detected and treated [2]. Colonoscopy is the established method of choice and gold standard for detection and removal of colorectal polyps. However, the effectiveness of this procedure is jeopardized by the number of polyps missed depending upon the colonoscopist’s skill level, polyp size and morphology, location in the colon, bowel preparation, and other operator- and procedure-dependent factors [3]. Studies have reported that between 6% and 28% of present colorectal polyps are missed during colonoscopy [4], and as a result, they could develop into post-screening CRC or interval CRC, which amount to 5% to 8% of all CRCs [5]. In patients with inflammatory bowel disease (IBD), interval CRCs can even account for up to 50% of CRCs [6]. A study by le Clercq et al. reported that 86.4% of interval CRCs could be explained by procedural factors, including 57.8% of interval CRCs caused by missed polyps during colonoscopy, 19.8% caused by inadequate examination, and 8.8% caused by incomplete removal of polyps [7]. A study on interval CRCs in Sweden reported that individuals with interval CRCs had shorter survival times than individuals with CRCs detected in colonoscopy and that the hazard ratios for men with interval CRCs were 2.75 with 2.00 for women [8]. Macken et al. also report worse survival for patients with interval CRCs than those without: 80% of the patients with interval CRCs survived for 1.6 years compared with 2.8 years for patients without; 60% of the patients with interval CRCs survived for 4.7 years compared with 6.7 years for patients without interval CRCs [9]. Many technologies have been developed to reduce interval CRCs and to improve the quality of colonoscopy screening, including high-definition imaging; narrow-band imaging (NBI); magnification endoscopes; and more recently, deep-learning-based diagnostic aid.

Deep learning has been applied to solve complex problems in a variety of scientific domains including the biological and medical sciences [10]. In particular, convolutional neural networks (CNNs) have displayed impressive results in image analysis tasks such as image recognition [11,12], object detection [13], and segmentation [14], outperforming humans in several cases, and they have been applied extensively to biomedical imaging problems with many hundreds of articles published in the scientific literature (e.g., [15,16,17,18,19] from our group alone). Image segmentation is especially important for analyzing medical images, as the segmented image can provide insights into the structure’s size, volume, and morphology and can help physicians with detection and diagnosis [20]. For instance, machine learning models have been developed to segment and classify pulmonary nodules in chest scan datasets, assisting physicians in early-stage lung cancer diagnosis [21]. Machine learning and deep learning approaches have also been proposed to help improve colonoscopists’ skill level in detecting polyps during screening colonoscopy [22,23,24,25], and several clinical trials have investigated their effectiveness [26,27,28].

The use of deep learning for diagnostic aid in colonoscopy falls into one of three categories: (1) predicting polyp pathology, such as Li et al.’s work [29] classifying images of polyps into sessile serrated adenomas (SSA) and non-SSA, or Byrne et al. [30], who proposed a CNN model to classify neoplastic vs. non-neoplastic polyps. (2) The second category is polyp detection with bounding boxes, such as Shin et al. [2], who used region-based CNN (R-CNN) [31] for polyp detection with a processing time of 39 ms per frame, which is borderline too slow for processing colonoscopy videos in real time. Urban et al. [22] implemented a YOLO-derived [32] architecture for polyp detection in videos and achieved an accuracy of 96.4% with a processing time of 10ms per frame, making it possible to detect polyps with high accuracy in real time. (3) The third category is polyp segmentation at the pixel level of video frames, which is computationally more demanding. Examples are Brandao et al. [33], who proposed a fully convolutional neural network (FCN) with 51 ms prediction time per image, which is too slow for use during procedures, and other attempts such as Vazquez et al. [34] using Mask R-CNN [35] or the work by Jie et al. [36]—but none of these meet the real-time processing speed constraints. The work by Guo et al. summarized the performances of several polyp segmentation models on the widely used public CVC-ClinicDB [37] dataset [38], where a baseline ResUNet model obtained a dice coefficient of 79.55% [39], and an ensemble of multiple models, referred to as MED-Net, achieved a dice coefficient of 91.3% [40]. In a study by Mahmud et al., a modified encoder–decoder segmentation model referred to as PolypSegNet achieved a dice coefficient of 91.5% on CVC-ClinicDB and 88.7% on another commonly benchmarked public dataset, Kvasir-SEG [41]; however, the model only has a near-real time inference speed of 39 ms per image (25 frames per second) [42]. Tomar et al. implemented a feedback attention network FANet and achieved dice coefficients of 93.55% on CVC-ClinicDB and 88.03% on Kvasir-SEG [43]. However, the abovementioned segmentation models are all fully supervised and must train on pixel-level, hand-annotated masks, which are extremely time-consuming to obtain. To leverage unlabeled data in model training, Wu et al. proposed a semi-supervised polyp segmentation model with an adversarial learning method. When trained on 30% mask-labeled images and 70% unlabeled images, this model achieved dice coefficients of 89.29% on CVC-ClinicDB and 80.95% on Kvasir-SEG, and when trained on 15% mask-labeled images and 75% unlabeled images, the dice coefficient scores dropped to 82.18% on CVC-ClinicDB and 76.76% on Kvasir-SEG [44].

A real-time polyp segmentation model can be a major step toward automation of non-invasive procedures using capsule endoscopy [45], and in the future, full robotic automation of the entire screening procedure. However, most published segmentation approaches suffer from the drawback that prediction throughput is too low to be real-time-capable even with a GPU (graphics processing unit); furthermore, most segmentation models require pixel-level annotated training data, which is very labor-intensive to obtain. In this study, we propose a weakly supervised [46] U-Net model [47] that leverages easy-to-obtain bounding box annotations to predict pixel-level segmentation masks of polyps in real time. The main contributions of this work are (1) a model that can predict pixel-level segmentation masks despite using only bounding box annotation as supervision source during training and, thus, avoids the need for expensive data annotation and (2) a model can generate polyp segmentations in real time at 45 FPS and, thus, assist with real-world colonoscopy screening procedures. The weakly supervised model is trained through an iterative process, where the model iteratively refines its own training targets (imperfect predictions of polyp segmentation masks), while weak external feedback is used to guide the training process by ensuring that all intermediary predictions on training images are consistent with the corresponding bounding box annotations. This process demonstrably recovers the correct segmentation mask in almost all cases as the network is trained. The model is trained on the publicly available CVC-ClinicDB dataset and a novel Polyp-Box-Seg dataset of 4070 colonoscopy images obtained from over 2000 patients. We show that the iterative training process achieves the same performance level (dice coefficient and pixel-wise accuracy) as would have been obtained by supervised training on human-annotated segmentation masks. In addition, we find that pooling together all labeled data (bounding box annotations and segmentation masks) during training further improves the model’s accuracy. Various CNN models are evaluated on the CVC-ClinicDB dataset; the Kvasir-SEG dataset; and the Polyp-Box-Seg dataset, in particular, on the subset of sessile serrated adenomas (SSA) in Polyp-Box-Seg, which are the most challenging polyps to correctly identify and segment due to their morphology and appearance [29]. The Polyp-Box-Seg dataset, together with a real-time video demonstration of the segmentation system, are available at www.igb.uci.edu/colonoscopy/AI_for_GI2.html (accessed on 18 April 2022).

## 2. Materials and Methods

### 2.1. Deep Learning Architecture

**Figure 1:**
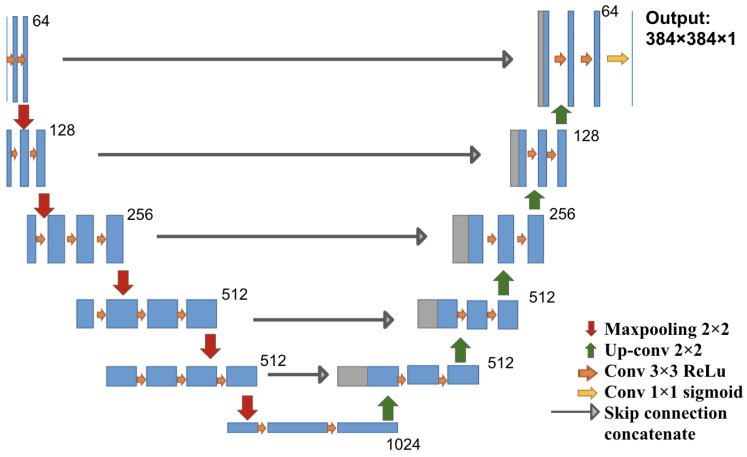
U-Net architecture: each block in the down-sampling path consists of convolution and max pooling operations, and each block in the up-sampling path consists of up-convolution and convolution operations. Each blue box corresponds to a multi-channel feature map. The number of features at the end of each block is denoted on top of the box. Arrows of different colors denote the different operations.

The architecture used in this study is a U-Net architecture, which is a fully convolutional network with a contracting path (encoder) and an expanding path (decoder), producing a segmentation prediction that is the same size as the input image. We chose to use five down-sampling blocks and five up-sampling blocks in the model, as shown in Figure 1. We used VGG16 weights trained on ImageNet as initial weights for the encoder component and random initial weights for the rest of the network [12]. For a detailed description of the U-Net architecture, please see Appendix A.

### 2.2. Iterative Weakly Supervised Training

To start the weakly supervised segmentation training process, initial segmentation masks for polyps in the training images must be generated, even if they are inaccurate. Three different approaches to obtain these masks were tested: (1) the initial mask for each polyp is approximated by a solid circle located in the center of the bounding box and sized to fill the box but not exceed any of its edges; (2) a segmentation model is pre-trained on the public polyp datasets, and its predictions serve as initial training targets after removing all parts of the predictions that exceed the annotated bounding boxes; and (3) a combination of approaches (1) and (2): initial masks are generated as in (2) using a pre-trained network, but if the predicted area within one bounding box occupies less than 30% of the box, then this initial prediction is rejected and replaced by a solid circle, as in (1). The third approach resulted in initial masks, of which approximately half were solid circles, as in (1), and half were predicted masks, as in (2).

These masks were then used as training targets for the corresponding input images in the iterative training scheme. After each iteration, the masks were updated using the network’s predictions. The bounding box annotations were used to define the weighted training loss function (as discussed in Section 2.3) and to remove all positive polyp predictions that fell outside of the bounding box by setting those mask values to 0. Then, the neural network was re-trained on the updated masks, and the process of updating masks and network was repeated for several iterations.

**Figure 2:**
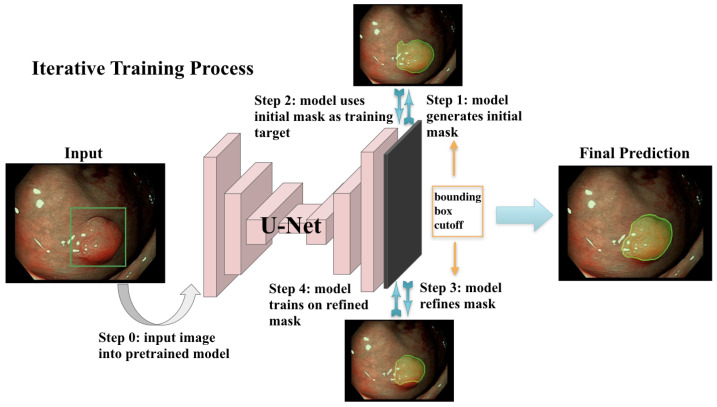
The model trains in an iterative scheme where it first generates an initial mask (Step 1) from input polyp image (note that the real bounding box is not shown to the network; only the box coordinates are used); then, the network trains and updates its parameters based on the initial mask (Step 2). The updated network in turn predicts a refined mask which replaces the previous mask (Step 3). The network then trains on the updated mask (Step 4) and updates the model parameters. The masks and the network are updated iteratively until training terminates.

This iterative training process is illustrated in Figure 2. The model was trained with a batch size of 12 for 3 epochs per iteration for the first 2 iterations and then for 6 epochs for another 6 iterations. The initial learning rate was 0.0001 for the first two iterations and decay with a factor of 0.0005 at each epoch starting from the third iteration. The Adam optimizer was used for training. Training was terminated after the validation loss stopped improving for two consecutive iterations.

The dice coefficient (F1 score), accuracy and confusion matrix are used as metrics to evaluate model performances [48]. For a detailed description of the metrics, please see Appendix B.

### 2.3. Masked Loss Function

Using this weakly supervised training procedure entails that the training targets are sometimes not a perfect representation of the polyps’ outlines, especially in the early iterations. To reduce the impact of these inaccuracies on the neural network performance, we used a pixel-wise weighted loss function with the pixel weights accounting for our confidence on the most likely location of the polyps within a bounding box. The loss mask is implemented using oval shaped rings, each extending to the four edges of its corresponding bounding box. The ring has a value of zero along its length, while its center and surrounding area have a mask value of one, with a gradual transition between the two regimes. The effect of this construction is that the neural network’s predictions are heavily scrutinized only in areas outside of the bounding boxes (which are guaranteed to be free of polyps) and areas in the center of the bounding boxes (which are guaranteed to belong to a polyp), while the model’s predictions in the areas in between (close to the bounding box’s edges) are left mostly untouched. Please note that the bounding boxes and loss were only used during training, and neither are present or used when the system is in live operation on videos or test images.

Mask M (as obtained by Algorithm 1), DNN prediction P, and Label Y all have dimensions of (batch_size × image_width × image_height).

$$Masked\_Loss\left(P,Y,M\right)=\frac{1}{\sum M}\sum \left(M⊙(binary\_crossentropy)(Y,P)\right)$$

**Algorithm 1** Loss mask for semi-supervised training.**Require:** bounding box width/height/coordinates: $box_{w},\;box_{h},\;center_{x},\;center_{y}$ **Require:** image width/height: w,h inner_radius $\leftarrow min(box_{w},\;box_{h})\times inner\_ring\_diameter\times 0.5$ outer_radius $\leftarrow max(box_{w},\;box_{h})\times outer\_ring\_diameter\times 0.5$ x_stretching $\leftarrow box_{w}\times 1/box_{h}\;if\;box_{w}\geq box_{h}\;else\;1$ y_stretching $\leftarrow box_{h}\times 1/box_{w}\;if\;box_{h}\geq box_{w}\;else\;1$ $Y,X\leftarrow {\left[1,\ldots ,h\right]}^{T},\left[1,\ldots ,w\right]$ $dist\_from\_center\leftarrow \sqrt{{\left(X-center_{x}\right)}^{2}/x\_stretching+{\left(Y-center_{y}\right)}^{2}/y\_stretching}$ inv_mask ←(dist_from_center<outer_radius)×(inner_radius<dist_from_center) ramp $\leftarrow {\left|dist\_from\_center-(outer\_radius+inner\_radius)/2\right|}^{1.5}\times \;inv\_mask$ mask ←ramp/max(ramp)+(1−inv_mask) **return**
mask

### 2.4. Dataset and Preprocessing

Four datasets were used for training and/or evaluation: (1) the ImageNet competition dataset, on which the VGG16 weights were trained [49]; (2) the public CVC-ClinicDB dataset of 612 polyp images with ground truth segmentation masks; (3) the public Kvasir-SEG dataset of 1000 polyp images with ground truth segmentation masks and bounding box coordinates of the polyp regions; and (4) a novel dataset, Polyp-Box-Seg, of 4070 images containing unique polyps collected from over 2000 patients. In the Polyp-Box-Seg dataset, all images with a polyp were annotated with bounding boxes over the polyp region, and a randomly chosen subset of 1300 polyp images was further annotated with segmentation masks by human experts. The set of 4070 polyp images was hand-selected from colonoscopy screening videos so that each image contains an unique polyp. This is to prevent correlation between images and thus to avoid a possible intra-patient polyp similarity bias. The Polyp-Box-Seg dataset contains polyps of all sizes and morphologies and covers all portions of the colorectum. Locations and dimensions of bounding boxes over the polyp regions were recorded by a team of colonoscopists. The original resolution of these images is 480 × 640 pixels, which was resized and padded to dimensions of 384 × 384. The data were normalized by subtracting its mean pixel value and by dividing by its standard deviation before training. While modern scopes generally operate at a higher resolution than 384 × 384, a prior study by Urban et al. [22] found that changes in input resolution have an almost negligible effect on automated polyp detection accuracy (comparing a resolution of 480 × 480 to 224 × 224 pixels). The dataset contains polyps of all histologies shown in Table 1, with a total of 349 sessile serrated adenomas (SSA), which is the polyp type that is the most difficult to delineate and annotate. Out of those 349 images, 90 have human labeled segmentation masks (chosen at random). Data augmentation techniques that were applied during training include (1) random translations of the image in any direction by up to 60 pixels but limited to never move parts of a polyp outside of the image; (2) zooming in or out of the images by up to +/−10%; (3) random mirroring of the image (horizontal and vertical); and (4) color augmentations performed by shifting each of the three color channels separately using a Gaussian distribution (mean 0 standard deviation 0.1) and thus slightly changing the images’ overall hue.

### 2.5. Model Training

**Figure 3:**
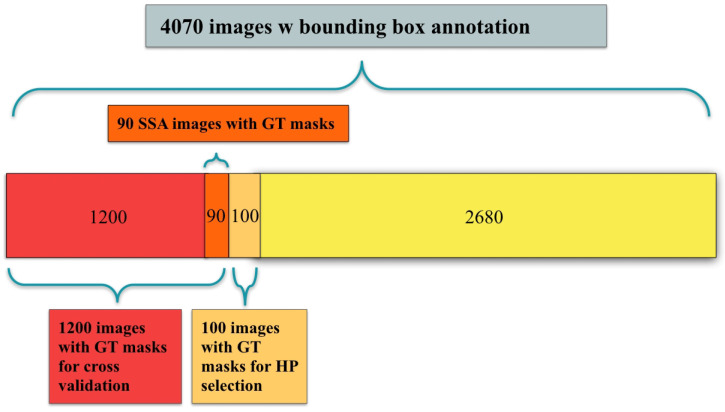
A breakdown of the 4070 polyp-containing images in training and testing: all 4070 images contain bounding box annotations of polyps, and a subset of 1300 images additionally contains human labeled ground truth (GT) segmentation masks. Among the 1300 images, 100 are used for hyperparameter (HP) selection and 1200 are used for model evaluation using cross-validation; 90 out of the 1200 images are sessile serrated adenomas (SSA).

An U-Net model was constructed as depicted in Figure 1 and initialized as described in Section 2.6. The following experiments were conducted:

1. A fully supervised model was trained on the 612 polyp images and segmentation masks of the public CVC-ClinicDB dataset. The weights of this model were used as a starting point for further experiments. This model is denoted as Full-Sup-1-VGG.
2. A fully supervised model was trained and evaluated via 10-fold cross-validation on the 1300 polyp images with segmentation masks from the Polyp-Box-Seg dataset. While the entire dataset contains 1300 images with segmentation masks, 100 of those were randomly selected and reserved for hyperparameter selection, with the 10-fold cross-validation being performed on the remaining 1200 images. A detailed breakdown of the data subsets used can be found in Figure 3. Weights pre-trained on the CVC-ClinicDB dataset were used as initial weights for this model. This model is denoted as Full-Sup-2. The cross-validation procedure trains 10 independent models and tests each on a different set of 120 images. This approach both significantly reduces the variance of the estimated (average) test score compared with a naïve single train-test data split and allows for an estimation of the variance of test accuracy estimates.
3. A weakly supervised model was trained using the bounding box annotations on the 4070 polyp images from the Polyp-Box-Seg dataset in a 10-fold cross-validation, as described in Section 2.2. Three approaches to generating the initial segmentation targets were evaluated:
  a All initial training targets are set to be a solid circle in the center of the bounding boxes, with a diameter equal to 4/5 of the box’s shorter side (width or height). The area outside of the bounding boxes is thereby assumed to not contain any polyps and serves as “background” category. This model is referred to as Weak-Sup-Box-CI in the following (Circular Initialization).
  b Predictions of the model trained on the public CVC-ClinicDB dataset are used as initial targets. All pixels with a predicted probability over 0.5 that lie within a bounding box are assumed to show part of a polyp, while all other pixels are considered to be background pixels. Models trained on this initialization method will be denoted as Weak-Sup-Box-PI (Prediction Initialization).
  c The same initialization scheme as in b is used, but bad predictions are replaced with a solid circle as in a. Polyp predictions are considered bad if they occupy less than 30% of the bounding box area. Models trained using this initialization method are denoted as Weak-Sup-Box-HI (Hybrid Initialization). Weak-Sup-Box-HI is evaluated on the Kvasir-SEG dataset.
4. A segmentation model was trained using the 1300 images with human segmentation labels together with the 2770 images with bounding boxes (a total of 4070 images) from the Polyp-Box-Seg dataset. Initial masks for the 2770 images with bounding boxes were generated in the hybrid manner, as described in c, and these masks were updated at the end of each training iteration, as described in Section 2.2. Segmentation mask labels were used for the 1300 images throughout training without being iteratively updated, as these labels are already accurate. In each iteration, the model trains on the mixture of 4070 images from both supervision sources, and at the end of the iteration, the model updates the masks for the 2770 images using model prediction. Models trained with a combination of weakly supervised bounding box targets and fully supervised segmentation mask targets are denoted as Weak-Sup-Mix.
5. A Weak-Sup-Mix model was trained as described in 4 and evaluated on sessile serrated adenomas using 10-fold cross-validation.
6. A Weak-Sup-Mix model was trained as described in 4 and evaluated on CVC-ClinicDB using 10-fold cross-validation. In each fold, the model was trained on all images from the Polyp-Box-Seg dataset plus 90% of the CVC-ClinicDB data and validated on the remaining 10% of the CVC-ClinicDB dataset. In the first two iterations, one extra training epoch on CVC-ClinicDB data was added at the end of three training epochs on all data, and in the remaining six iterations, two extra training epochs on CVC-ClinicDB data were added at the end of six training epochs on all data.
7. A fully supervised segmentation model was trained and evaluated using 10-fold cross-validation on the CVC-ClinicDB dataset using initial weights pre-trained on the Weak-Sup-Mix model, as described in 4. This model is named Full-Sup-3. This pre-trained model essentially transfers knowledge of polyp shapes learned on the Polyp-Box-Seg dataset to the CVC-ClinicDB dataset, where it is fine-tuned to adjust to the differences between the datasets (such as different cameras and lighting conditions). The model was trained for 30 epochs with an initial learning rate of 1 × 10^−4^, learning rate decay of 5 × 10^−4^ after each epoch, and a batch size of 1.

### 2.6. Network Initialization

Neural networks are commonly initialized with small random weights before training, but it has been common practice to instead use weights from networks trained on broadly related tasks, as this is extremely beneficial. In this work, all models with suffix “-VGG” were initialized with weights from a VGG16 model trained on the ImageNet dataset of 1.2 million images. Although that dataset does not contain medical polyp images, it has been shown repeatedly in the past that this transfer learning process greatly aids subsequently trained polyp detector models. Other models were initialized with weights trained from Full-Sup-1-VGG. Different initializations allow us to assess the importance of the public polyp dataset on which Full-Sup-1-VGG was trained.

## 3. Results

### 3.1. Pre-Trained Weakly Supervised Models on Polyp-Box-Seg

A series of models pre-trained on the CVC-ClinicDB dataset are trained and evaluated on the Polyp-Box-Seg dataset. First, as a baseline, a fully supervised model called Full-Sup-2 is trained on the 1300 segmentation masks annotated images from Polyp-Box-Seg alone. Full-Sup-2 reaches an average dice coefficient of 81.52% and a pixel-level accuracy of 98.76% using 10-fold cross-validation (Full-Sup-2 in Table 2).

Second, a series of weakly supervised models are trained using bounding box annotations alone (4070 images from Polyp-Box-Seg), with three different segmentation mask initialization methods (circle, prediction, and hybrid). With the same 10-fold cross-validation, these models achieve average dice scores of 77.58 ± 0.66% (Weak-Sup-Box-CI in Table 2), 77.78 ± 0.87% (Weak-Sup-Box-PI in Table 2), and 81.36 ± 0.43% (Weak-Sup-Box-HI in Table 2). The Weak-Sup-Box-HI model performs significantly better than the two others, which highlights that the quality of the initial training targets is of significant importance and that the iterative training process cannot compensate for this entirely. The Weak-Sup-Box-HI model, trained iteratively on 4070 bounding box annotations, achieves a comparable pixel-level segmentation performance with the fully supervised Full-Sup-2 model (dice coefficient 81.52 ± 0.41%), which is trained on 1300 manually labeled segmentation masks. There is no significant difference between the performances of those two models according to a *t*-test (*p*-value = 0.87). This shows that, even though bounding box annotations lack pixel-level information on the shape of polyps, we can achieve competitive results on the pixel-wise segmentation task using the iterative weakly supervised training procedure.

Third, the weakly supervised model Weak-Sup-Mix in Table 2 is trained using both annotations combined, i.e., 1300 images with segmentation masks and 2770 images with bounding box annotations. The iterative weakly supervised procedure is applied to the 2770 images (the annotations for the 1300 images are already correct and are excluded from being changed during this procedure). This model reaches a dice score of 85.53 ± 0.33%, significantly surpassing the performance of all other models, including Full-Sup-2, which was trained on segmented masks and reaches a dice coefficient of only 81.52 ± 0.41% (*t*-test *p*-value of 0.00096 < 0.05). This suggests that training with the maximum amount of data and heterogeneous annotations works better than utilizing either single supervision source on its own.

**Figure 4:**
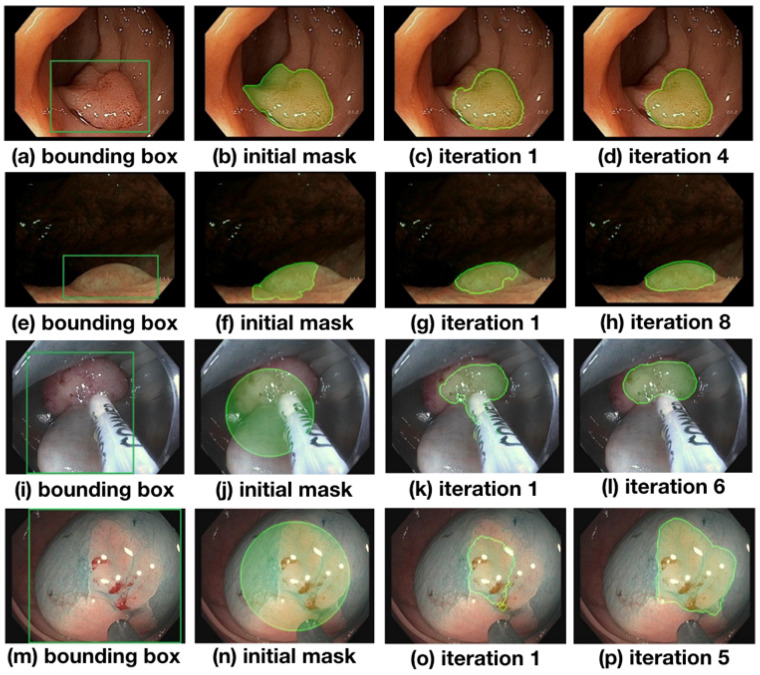
Evolution of the segmentations starting from their initialization state over the course of training. Masks gradually approach the ground truth label as training progresses. The first column shows images with the bounding box annotations that are used to guide the learning process. The second column shows the initial set of masks generated with different approaches: images (**b**,**f**) use model prediction as initial masks, while images (**j**,**n**) use circles as initial masks.

An evolution of the segmentation masks predicted by the network is shown in Figure 4 and discussed in the following. At the beginning of the training process, the initial masks are far from perfect (see Figure 4b,f,j,n) but improve significantly over the course of training the network. In Figure 4, (b) and (f) depict masks initialized with predictions made from Full-Sup-1-VGG, a model pre-trained on the public CVC-ClinicDB polyp dataset (see (b)). Differences between CVC-ClinicDB and our dataset reduce this model’s segmentation accuracy on our dataset, causing the initial segmentation masks in (b) and (f) to not to overlap with the true polyps very well. Nevertheless, several iterations of the weakly supervised training scheme allow the model to segment polyps accurately (see (d) and (h) in Figure 4). The last two rows of Figure 4 show masks that are initialized with circles (images (j) and (n); see (a) for details), and despite these poor initial labels, the model still gradually recovers the contour of the polyps close to ground truth during training ((l) and (p) in Figure 4). Notice that image (i) contains a surgical instrument in the bounding box area that is initially part of the circular labels and falsely predicted in an early iteration (j) but correctly excluded from the predicted mask after six iterations of training. Compared with the bounding boxes in (a) and (e), which are tightly placed around the polyps, bounding boxes in (i) and (m) contain a large non-polyp area. Nevertheless, even with loosely placed bounding boxes where the polyp center, size, and shape are harder to determine, the model eventually finds the polyp contour.

To compare the effectiveness of the four weakly supervised models from Table 2, we use a fixed validation set and compute the average number of true positives (TP), true negatives (TN), false positives (FP), and false negatives (FN) in their predictions (Table 3). The performances between Weak-Sup-Box-PI and Weak-Sup-Box-CI are similar, with Weak-Sup-Box-PI having a higher TP and higher FP. Weak-Sup-Box-CI yields the highest number of FN among the four models. This suggests that simply assuming polyps to be circles is not enough for initial mask generation. Weak-Sup-Box-HI is more effective than the first two models because it generates better initial masks by using the hybrid method. When trained on the combination of bounding-box-labeled data together with mask-labeled data, the Weak-Sup-Mix model is the most effective among these four weakly supervised models and reaches the highest TP and lowest FP in these predictions. This shows the benefit of effectively pooling all images together in training, even when their supervision sources are different.

### 3.2. Weakly Supervised Models Initialized with VGG16 Weights on Polyp-Box-Seg

Models initialized with VGG16 weights in Table 4 performed slightly worse than those that benefited from pre-training on the CVC-ClinicDB dataset. However, even under these circumstances, the model with the addition of weakly supervised training (Weak-Sup-Mix-VGG in Table 4) performed significantly better than the model trained on only the human segmentation annotations (Full-Sup-2 in Table 2), with a dice coefficient of 85.08 ± 0.60% compared with only 81.52 ± 0.41% (*t*-test *p*-value of 0.0018 < 0.05).

### 3.3. Weakly Supervised Models Tested on Sessile Serrate Adenomas Alone

The best-performing weakly supervised model, Weak-Sup-Mix, which utilizes both bounding boxes and human annotations, is evaluated on sessile serrated adenomas (SSA), the most difficult category of polyps to segment correctly. Weak-Sup-Mix reaches a dice score of 84.56 ± 2.38% on SSA segmentation, as in Table 5. The same model, but initialized with VGG16 weights instead of pre-training on the CVC-ClinicDB data (Weak-Sup-Mix-VGG), reaches a dice score of 83.66 ± 1.95%. As one would expect, the segmentation accuracy on SSA is worse than that on an average polyp, but it turns out that the difference in dice scores is only 1.0% between those two groups (*t*-test *p*-value 0.77).

### 3.4. Weakly Supervised Models on CVC-ClinicDB

The model Weak-Sup-Mix-VGG is initialized with VGG16 weights and trained on bounding boxes annotation and human-annotated images and achieves an average dice score of 90.43 ± 0.43% using 10-fold cross-validation on the CVC-ClinicDB dataset (Weak-Sup-Mix-VGG on CVC in Table 6). Full-Sup-3 in Table 6 is the fully supervised model trained and evaluated on the CVC-ClinicDB dataset with 10-fold cross-validation. This model uses the weights of a fully trained Weak-Sup-Mix-VGG model as initial weights (see details in 4). This transfer learning procedure allows Full-Sup-3 to utilize features learned from the Polyp-Box-Seg dataset and to obtain an average dice score of 91.79 ± 0.43% on the CVC-ClinicDB data, surpassing the best test score of 91.3% reported for MED-NET [40] as well as 91.5% reported for PolypSegNet [42].

Indeed, the current best-performing models on the CVC-ClinicDB dataset include MED-NET (dice coefficient 91.3%), PolypSegNet (dice coefficient 91.5%), and FANet (dice coefficient 93.55%) [40,42,43]. However, several issues with these three approaches must be considered. First, the images in the CVC-ClinicDB dataset are highly correlated with each other, as they correspond to consecutive video frames. In contrast, the Polyp-Box-Seg dataset contains 4070 unique polyp images, and thus, these images are highly uncorrelated with each other. Additionally, CVC-ClinicDB contains ordinary and clean polyps with few artifacts, while the Polyp-Box-Seg data includes challenging polyps with additional features such as forceps, snares, debris, and fluid, to name a few. Second, the results reported for MED-NET and FANet are obtained on a fixed test set containing 20% of the CVC-ClinicDB dataset, without varying this test set and without doing any systematic cross-validation experiments. In all our experiments, we see differences in test scores of up to 3% using different, randomly selected, test sets. Thus, the reported results come with no error bars and could provide overly optimistic performance estimates. And finally, these models have relatively larger inference times due to their complex architectures. For instance, MED-NET consists of an ensemble of several deep encoder–decoder models, which significantly increases its inference time, preventing deployment on video data and real-time applications. While PolypSegNet is faster and can process up to 25 frames per second, this is barely enough for video processing, and slower than the models presented here.

### 3.5. Weakly Supervised Models on Kvasir-SEG

The model Weak-Sup-Box-HI uses weights were pre-trained on CVC-ClinincDB data as initial training weights (as described in c) and therefore cannot be evaluated on the CVC-ClinicDB dataset. This model is instead evaluated on the public Kvasir-SEG dataset using a random 90/10 training/testing split. Weak-Sup-Box-HI uses bounding box annotations as the only source of supervision and trains in the iterative manner. This weakly supervised model achieves a dice coefficient of 82.81% on the testing set, as shown in Table 7. The fully supervised ResUnet model used in the Kvasir-SEG paper achieved a dice coefficient of 78.77% on the testing set [41]. A semi-supervised model proposed by Wu et al. achieved a dice coefficient of 80.95% when training with 30% mask-labeled images and 70% unlabeled images [44]. Compared with one of the current best-performing models, PolypSegNet, which has a 88.7% dice coefficient testing on Kvasir-SEG, Weak-Sup-Box-HI reaches a competitive score with a faster inference time and only requires bounding box labels for training.

All of the trained models above use the same underlying U-Net architecture, which is capable of processing video data at 45 frames per second (fps) using a Titan RTX GPU. Thus, these models can be easily deployed in real-time colonoscopy screenings, even when using a cheaper consumer-grade GPU. A video demonstrating real-time polyp segmentation in colonoscopy can be found at www.igb.uci.edu/colonoscopy/AI_for_GI2.html (accessed on 18 April 2022).Training and validation losses for each model, and the confusion matrix for each model predictions can be found in supplementary Tables S1 and S2.

## 4. Discussion

Several convolutional neural networks were applied to the task of real-time polyp segmentation using either only bounding box annotations as supervision source and/or human annotations of the polyp contours. Bounding box annotations are relatively easy to obtain but contain much less information compared to human-labeled segmentation masks. Nevertheless, our proposed iterative weakly supervised training procedure enables training models on bounding boxes that reach a competitive performance level (81.36% dice coefficient) compared with fully supervised models trained on human-labeled segmentation masks (81.52% dice coefficient). Moreover, our combined training approach on human-labeled segmentation masks and bounding box annotations further improves the model’s polyp segmentation accuracy (85.53% dice coefficient). The proposed model performs well in the most challenging cases corresponding to the segmentation of sessile serrated adenomas (84.56% dice coefficient), and when tested on the public CVC-ClinicDB, our model is comparable with other state-of-the-art approaches. Furthermore, our models can process video data at 45 fps and thus can be easily deployed in real-world colonoscopy videos.

The proposed weakly supervised training algorithm can greatly reduce the cost of annotating large volumes of data by human experts, thus resolving a major limitation in obtaining large amounts of training data for semantic segmentation. Instead of annotating segmentation masks for all images, researchers can simply use bounding box annotations as supervision source or only annotate segmentation masks for a small fraction of images and can train models on a mixture of ground truth masks and bounding box annotations.

Computer vision for object detection entails a hierarchy of at least four different tasks: (1) the detection of an object (presence/absence), (2) its localization (bounding box), (3) its segmentation (contour), and (4) its representation in three dimensions (3D model). In a previous study, we tackled the problem of using deep learning for real-time polyp detection and localization with bounding boxes in colonoscopy videos [22]. The newly proposed system extends this approach to the prediction of detailed pixel-level segmentations of polyps in real time, while also avoiding the problem of increased human annotation effort. Thus, the first three problems in the hierarchy are largely solved. A possible future extension is to tackle the fourth problem and to develop a multi-view stereo (MVS) model to reconstruct the 3-dimensional structure of any polyp: for instance, by combining the current system’s predicted 2D segmentations with depth estimation derived from multiple viewpoints of the same polyp [50,51]. Whether the problem can be solved without using multiple viewpoint polyp images or using the limited and constrained multiple viewpoints provided by existing colonoscopy video frames, and whether it requires new carefully acquired data are open questions that need to be investigated.

## 5. Conclusions

To date, several AI-based tools have been designed to improve the quality of colonoscopy and to reduce the rate of post-screening CRCs by reducing the amount of polyp missed during screening. The proposed polyp segmentation approach can assist colonoscopists by drawing contours around polyps in real time. It also brings the technology one step closer toward partially or even fully automated colonoscopy, especially in the context of capsule colonoscopy, where it could be used to detect polyps and to generate automated reports. These reports could include size and volume information to guide clinical interventions, such as automated surgical polyp excision.

## Figures and Tables

**Table 1:** Histology information for 4070 polyp images from the Polyp-Box-Seg dataset.

Histology	Count
Tubular adenoma	2102
Hyperplastic	909
Sessile serrated adenoma	349
Non-serrated sessile	446
Tubulovillous adenoma	64
Inflammatory	42
Traditional serrated adenoma	33
Lymphoid nodule	19
CA adenocarcinoma	14
Hamartomatous	11
Juvenile polyp	6
CA lymphoma	5
Sessile serrated adenoma w dysplasia	3
Mucosal prolapse	3
CA squamous/epidermoid	1
Other	63

**Table 2:** Average test-set scores and standard deviation using 10-fold cross-validation on the Polyp-Box-Seg images. The neural networks in this table are pre-trained on the public CVC-ClinicDB dataset and then further trained as described in the text.

	Full-Sup-2	Weak-Sup-Box-CI	Weak-Sup-Box-PI	Weak-Sup-Box-HI	Weak-Sup-Mix
Dice Coefficient	81.52 ± 0.41%	77.58 ± 0.66%	77.78 ± 0.87%	81.36 ± 0.43%	**85.53 ± 0.33%**
Accuracy	98.76 ± 0.09%	98.42 ± 0.10%	98.50 ± 0.11%	98.67 ± 0.09%	**98.96 ± 0.07%**

**Table 3:** Confusion matrices from weakly supervised models’ predictions on one split of the validation set.

	Weak-Sup-Box-CI	Weak-Sup-Box-PI	Weak-Sup-Box-HI	Weak-Sup-Mix
TP	5039.01	5947.08	6180.07	**6333.31**
TN	139,604.96	139,414.03	139,341.57	**139,755.01**
FP	512.68	703.62	776.07	**362.63**
FN	2299.32	1391.26	1158.26	**1005.02**

**Table 4:** Average test-set scores and standard deviation using 10-fold cross-validation on the Polyp-Box-Seg images. Models are initialized with VGG16 weights and then trained as described in the text.

	Full-Sup-2-VGG	Weak-Sup-Box-CI-VGG	Weak-Sup-Mix-VGG
Dice Coefficient	79.11 ± 0.93%	76.14 ± 0.67%	**85.08 ± 0.60%**
Accuracy	98.56 ± 0.14%	98.31 ± 0.08%	**98.93 ± 0.09%**

**Table 5:** Average test-set scores and standard deviation using 10-fold cross-validation on the 90 sessile serrated adenoma images with human-labeled segmentation masks.

	Weak-Sup-Mix on SSA	Weak-Sup-Mix-VGG on SSA
Dice Coefficient	**84.56 ± 2.38%**	83.66 ± 1.95%
Accuracy	**98.23 ± 0.34%**	98.05 ± 0.39%

**Table 6:** Average test-set scores and standard deviation using 10-fold cross-validation on CVC-ClinicDB from the Weak-Sup-Mix-VGG model and from the Full-Sup-3 model as described in text.

	Weak-Sup-Mix-VGG on CVC	Full-Sup-3 on CVC
Dice Coefficient	90.43 ± 0.43%	**91.79 ± 0.43%**
Accuracy	98.87 ± 0.08%	**99.06 ± 0.07%**

**Table 7:** Test-set scores on Kvasir-SEG from the Weak-Sup-Box-HI model.

	Weak-Sup-Box-HI on Kvasir-SEG
Dice Coefficient	82.81%
Accuracy	95.41%

## Data Availability

The data will be made public upon acceptance of manuscript. Otherwise, please contact us for the data.

## Supplementary Materials

The following supporting information can be downloaded at https://www.mdpi.com/article/10.3390/jimaging8050121/s1. Table S1: Training and validation losses for each model on a validation set; Table S2: Confusion matrix for each model predictions on a validation set.

## Appendix A

Below is a detailed description of the U-Net architecture used in the manuscript: The first down-sampling blocks consisted of two 3 × 3 × 64 padded convolutions followed by a rectified linear unit (ReLU) and a 2 × 2 max pooling operation with stride 2. At each down-sampling step, the number of feature channels was doubled. The second down-sampling blocks consisted of two 3 × 3 × 128 padded convolutions followed by a rectified linear unit (ReLU) and a 2 × 2 max pooling operation with stride 2. The following three down-sampling blocks consisted of three 3 × 3 padded convolutions with numbers of feature channels of 256, 512, and 512, each followed by a rectified linear unit (ReLU) and a 2 × 2 max pooling operation with stride 2. Each of the up-sampling block consisted of a 2 × 2 up-convolution, a concatenation with the feature map from the down-sampling path, and two 3 × 3 convolution, each followed by a ReLU. At each up-sampling step, the number of feature channels was halved and concatenated with an equal number of feature channels from the corresponding block from the down-sampling path. The final layer used a 1 × 1 convolution followed by a sigmoid activation to map the 64 feature maps to a probability between 0 and 1. The closer to 1, the more likely this pixel contained polyp, and the closer to 0, the more likely this pixel contained background.

## Appendix B

The dice coefficient is a score measuring the similarity between the predicted mask and the ground truth mask label. The dice coefficient ranges between 0 and 1, with 0 indicating no spatial overlaps between the predicted and the ground truth masks and 1 being a perfect overlap between them. The accuracy score measures the percentage of correctly predicted pixels in the prediction mask compared with the ground truth. The confusion matrix contains several statistics, including the number of true positives (TP), true negatives (NP), false positives (FP), and false negatives (FN) in the predicted mask compared with the ground truth. Below is the mathematical formula for the dice coefficient and accuracy scores. The mathematical expressions for the dice coefficient and the accuracy are as follows.

$$dice=\frac{2\times Area_{gt}\cap Area_{predicted}}{Area_{gt}+Area_{predicted}}$$

$$accuracy=N_{correct}/N_{total}$$
